# Preoperative predictors of persistent enteropathic diarrhea after one-anastomosis gastric bypass in patients with type 2 diabetes mellitus

**DOI:** 10.3389/fsurg.2026.1901971

**Published:** 2026-08-12

**Authors:** Ilkhom B. Khayitov, Akhmadjon B. Babajonov, Saidislomxon S. Sayidburxonov

**Affiliations:** 1 Department of Surgical Diseases in Family Medicine, Tashkent State Medical University, Tashkent, Uzbekistan; 2 Department of Surgical Sciences, Central Asian Medical University, Fergana, Uzbekistan

**Keywords:** bariatric surgery, HbA1c, one-anastomosis gastric bypass, postoperative diarrhea, risk prediction, steatorrhea, type 2 diabetes mellitus

## Abstract

**Background:**

Persistent enteropathic diarrhea is a quality-of-life-limiting complication after one-anastomosis gastric bypass (OAGB). Patients with type 2 diabetes mellitus (T2DM) may be especially vulnerable owing to subclinical diabetic enteropathy and reduced pancreatic exocrine reserve, but no validated preoperative tool identifies them. We aimed to identify and internally validate routine preoperative predictors of persistent enteropathic diarrhea 12 months after OAGB in a T2DM cohort.

**Methods:**

In this single-center prospective cohort of 87 consecutive patients with T2DM who underwent primary OAGB (January 2020 – December 2024) and completed ≥ 12 months of follow-up, the primary outcome was persistent enteropathic diarrhea, defined as loose or watery stools at all three of the 3-, 6-, and 12-month visits. Preoperative HbA1c, serum albumin, C-reactive protein, coprogram findings, T2DM duration, and antidiabetic regimen were assessed by univariate and multivariable logistic regression, with internal validation by bootstrap resampling.

**Results:**

Persistent enteropathic diarrhea occurred in 26 of 87 patients (29.9%, 95% CI 20.5–40.6%). Three preoperative variables were independently associated with the outcome: HbA1c (OR 2.28 per 1%, 95% CI 1.53–3.73), steatorrhea on coprogram (aOR 4.36, 95% CI 1.21–16.82), and T2DM duration ≥ 10 years (OR 3.90, 95% CI 1.02–16.16). The multivariable model showed good calibration and discrimination, with an area under the receiver operating characteristic curve (AUC) of 0.863, but HbA1c alone provided comparable performance (AUC 0.827) with no significant additional gain from the full model. At a Youden-optimal HbA1c threshold of ≥ 8.25%, the negative predictive value reached 95.2%.

**Conclusion:**

In T2DM patients undergoing OAGB, preoperative HbA1c is a robust, clinically accessible single-variable predictor of persistent enteropathic diarrhea at 12 months; coprogram steatorrhea and long-standing diabetes add complementary pathophysiological context. These routine metrics can inform preoperative counseling and risk-adapted postoperative surveillance, and warrant prospective external validation.

## Introduction

Obesity and type 2 diabetes mellitus (T2DM) now travel together in nearly every part of the world. International Diabetes Federation put the number of adults living with diabetes at 589 million in 2024 and projects 853 million by 2050, with type 2 disease responsible for most of the burden (1). Growth is fastest in middle-income regions, including Central Asia, where dietary change, urbanization and an aging population converge (1). For patients with severe or long-standing T2DM, medical therapy alone seldom achieves durable remission. Randomized trials with long follow-up have established metabolic and bariatric surgery as the most effective treatment for sustained weight loss and improved glycemic control in this setting (2, 3).

Of the available metabolic procedures, one-anastomosis gastric bypass (OAGB), also known as mini-gastric bypass, has moved into the mainstream over the past decade and is now the third most commonly performed bariatric operation worldwide (4, 5). The technical appeal is straightforward: a single gastrojejunal anastomosis, shorter operating time than Roux-en-Y gastric bypass, and metabolic outcomes that are at least equivalent (4, 6). A recent network meta-analysis of 5-year randomized data identified OAGB as the most effective surgical option for sustained T2DM remission (7), and large observational series have reported higher 1- and 3-year remission rates (8).

Metabolic gains come at a cost. Persistent enteropathic diarrhea is one of the more clinically important post-OAGB gastrointestinal sequelae, affecting approximately one-quarter of patients in general OAGB cohorts (9). Its pathophysiology is multifactorial: prospective post-OAGB data document small intestinal bacterial overgrowth in approximately one-third and pancreatic exocrine insufficiency in approximately one-quarter of patients at six months (10), superimposed on the hypoabsorptive effect of the shortened common channel and altered enterohormonal signaling (11, 12). Nutritional deficiencies and malabsorptive symptoms occur more frequently after procedures with longer biliopancreatic limbs (6, 13). Beyond its impact on quality of life, persistent diarrhea contributes to electrolyte and micronutrient disturbance and, when refractory to medical management, may in a small subset of patients be a factor in revisional surgery (11). Two preoperative characteristics have particular pathophysiological relevance to this outcome. First, glycemic control: chronic hyperglycemia produces a well-characterized diabetic enteropathy involving both autonomic dysfunction (14) and impaired intestinal epithelial barrier integrity via GLUT2-dependent mechanisms (15); these underlying diabetic gastrointestinal alterations plausibly persist after bariatric surgery and may compound the malabsorptive effect of the procedure. Second, nutritional reserve, reflected in serum albumin: low preoperative albumin has been associated with adverse postoperative outcomes after bariatric surgery (16), and preoperative albumin has been identified as a predictor of postoperative hypoalbuminemia specifically after OAGB (17).

Chronic hyperglycemia drives a cluster of gastrointestinal disturbances, collectively called diabetic enteropathy, in which autonomic neuropathy, advanced glycation end-products, altered enteric motility and impaired intestinal barrier function combine to produce diarrhea, constipation or alternating bowel habits in up to three quarters of long-term diabetic patients (14, 15). After OAGB shortens the absorptive surface and changes transit and the microbiota, the clinical picture in diabetic patients can therefore differ from what is seen in non-diabetic recipients. So far, however, most reports on post-bariatric diarrhea have grouped diabetic and non-diabetic patients together, and dedicated analyses of T2DM cohorts after OAGB are scarce.

Several routinely measured preoperative parameters have a plausible link with postoperative enteropathic diarrhea. Glycated hemoglobin (HbA1c) summarizes three months of glycemic exposure and tracks the severity of diabetic autonomic neuropathy and intestinal barrier dysfunction (14, 15). Serum albumin reflects both nutritional reserve and systemic inflammation; preoperative hypoalbuminemia has emerged as one of the strongest modifiable predictors of serious bariatric complications in registries of more than half a million patients (16–18). C-reactive protein (CRP) captures the chronic low-grade inflammation typical of obesity and T2DM, and elevated preoperative levels have been associated with adverse outcomes after gastric bypass in diabetic patients (19). Coprogram findings such as steatorrhea or undigested fat may flag incipient maldigestion or pancreatic insufficiency that is otherwise silent (11, 18). Despite this biological coherence, no published study has formally evaluated these parameters together as a preoperative model for diarrhea after OAGB in a T2DM-only cohort.

Identifying high-risk patients before surgery would have several practical consequences: more individualized counseling, more thoughtful selection of biliopancreatic limb length, and structured postoperative surveillance with earlier work-up for treatable causes such as bacterial overgrowth and pancreatic insufficiency. The present study, in 87 consecutive patients with T2DM who underwent primary OAGB at a single tertiary center between 2020 and 2024, was designed with that aim. We examined the incidence of persistent enteropathic diarrhea at 12 months and evaluated HbA1c, serum albumin, CRP, coprogram findings and T2DM duration as preoperative predictors. To our knowledge this is the first study from Central Asia to address the question in a standardized T2DM cohort.

## Materials and methods

We carried out a single-center prospective cohort study of patients with T2DM who underwent primary OAGB at the Department of Surgical Diseases in Family Medicine, Tashkent State Medical University, Tashkent, Uzbekistan, between January 2020 and December 2024. Reporting follows the STROBE statement (20). Data were taken from a prospectively maintained departmental bariatric registry and, when needed, supplemented by manual review of paper and electronic records. Two investigators abstracted all variables independently, and any discrepancies were resolved by consensus.

### Ethical considerations

The study was approved by the Ethics Committee of Tashkent Medical Academy. The studies were conducted in accordance with the local legislation and institutional requirements. The participants provided their written informed consent to participate in this study.

### Patient selection

All consecutive patients aged 18 years or older with a confirmed diagnosis of T2DM who underwent primary OAGB during the study period were screened for eligibility. Indications for OAGB followed current international guidelines for metabolic and bariatric surgery, with diabetes-specific indications applied in line with the joint statement of the diabetes and surgical societies.

*Inclusion criteria:* Age ≥ 18 years; confirmed T2DM established at least six months before surgery; primary OAGB at our institution between January 2020 and December 2024; body mass index (BMI) ≥ 35 kg/m^2^ with obesity-related comorbidities or ≥ 40 kg/m^2^ without; complete preoperative laboratory data within seven days of surgery; and at least 12 months of postoperative follow-up.

*Exclusion criteria:* Patients were excluded for any of the following: a history of chronic diarrhea before surgery (loose or watery stools persisting for more than six months); known inflammatory bowel disease, celiac disease or irritable bowel syndrome; previous cholecystectomy or other gastrointestinal surgery; use of medications known to cause diarrhea (documented metformin-induced diarrhea, acarbose, orlistat or selective serotonin reuptake inhibitors, patients on metformin without medication-related diarrhea were eligible); documented postoperative dumping syndrome (Sigstad score ≥ 7); postoperative infectious diarrhea (positive stool culture or *Clostridioides difficile* testing); conversion or revision bariatric procedures during follow-up; or incomplete follow-up data at predefined assessment time points.

### Surgical technique

All operations were laparoscopic and performed by a single experienced surgical team using a standardized OAGB technique (4). Under general endotracheal anesthesia, with the patient in the supine French position with reverse Trendelenburg tilt, a standard five-port approach was used. Pneumoperitoneum was established at 12–14 mmHg. A long, narrow gastric pouch of approximately 120 mL volume and 18–20 cm length was created along the lesser curvature using sequential applications of a linear endoscopic stapler, calibrated over a 38-French bougie, beginning at the incisura angularis and continuing parallel to the lesser curvature up to the angle of His.

The biliopancreatic limb (BPL) was measured from the ligament of Treitz with a marked grasper at 5-cm increments and standardized according to the degree of obesity: 180 cm for patients with class II obesity (BMI 35 to 39.9 kg/m^2) and 200 cm for patients with class III or IV obesity (BMI at or above 40 kg/m^2). An antecolic, end-to-side gastrojejunostomy was created with a linear stapler, and the common enterotomy was closed in two layers using absorbable sutures. Methylene blue leak testing was routinely performed. Postoperative care. Venous thromboembolism prophylaxis with low-molecular-weight heparin was started 12 h after surgery and continued for three days. Early mobilization was initiated on postoperative day 1 and stepwise dietary progression over four to six weeks was supervised by the same clinical dietitian team, following the International Federation for the Surgery of Obesity nutritional guidelines. Standard long-term supplementation included oral multivitamins, vitamin B12, and iron in all patients; a proton pump inhibitor was prescribed on a symptom-driven basis only. Metformin and glucagon-like peptide-1 receptor agonists were not administered during the first postoperative year in any patient in the analysis cohort; patients whose diabetes management required these agents during the first postoperative year were excluded *a priori*.

### Predictor variables and preoperative assessment

All candidate predictor variables were measured preoperatively, within seven days of surgery, as part of the standard preoperative work-up. To preserve temporal validity, no postoperative measurements were used as predictors. Preoperative glycemic management was standardized and followed our institutional protocol, which targets a fasting plasma glucose ≤ 12 mmol/L on the day of surgery; this target was met in all included patients through adjustment of antidiabetic therapy. Laboratory analyses were performed in the central clinical laboratory of the institution using standardized commercial assays. Coprograms were assessed by trained clinical laboratory technicians under standard light microscopy of fresh stool samples; steatorrhea, undigested muscle fibers, mucus and leucocytes were each recorded as present or absent.

The primary candidate predictors were HbA1c (%), serum albumin (g/L), CRP (mg/L) and coprogram findings. Additional baseline variables collected were: demographics (age, sex); anthropometry (weight, height, BMI); diabetes-related variables (duration of T2DM, fasting plasma glucose, antidiabetic regimen); additional laboratory parameters (total protein, hemoglobin, total cholesterol); and selected comorbidities (arterial hypertension, osteochondrosis and sleep apnea).

### Outcome definition

The primary outcome was persistent enteropathic diarrhea, ascertained at the 12-month visit and defined operationally as documented loose or watery stools at each of the 3-, 6- and 12-month visits, with onset no earlier than one month after surgery. Patients with transient changes in bowel habit during the first postoperative month that resolved spontaneously were considered to be in the physiological adaptation phase and were not classified as having the primary outcome. Diarrhea attributable to identifiable competing etiologies [dumping syndrome [Sigstad score ≥ 7], infectious diarrhea [positive stool testing for Clostridioides difficile or enteric pathogens], or medication-induced diarrhea] was excluded as defined above.

### Follow-up protocol

Patients were scheduled for clinical follow-up at 1, 3, 6 and 12 months after surgery. At each visit a structured assessment was carried out, covering bowel habit (stool frequency over 24 h and stool consistency), anthropometry, glycemic control and laboratory follow-up as clinically indicated. The 12-month visit served as the primary outcome ascertainment point. All 87 patients included in the present analysis attended the 12-month visit, with full ascertainment of bowel-habit data at every scheduled visit.

### Statistical analysis

Statistical analyses were conducted in Python 3.11 (statsmodels, scipy, scikit-learn) and validated in R 4.3 (pROC, glm). Normal distribution was assessed using the Shapiro–Wilk test. Continuous variables are presented as mean ± SD or median (IQR) and compared via Welch's *t*-test or Mann–Whitney U test. Categorical data are shown as frequencies (%) and analyzed using Pearson's chi-squared (*χ*^2^) or Fisher's exact test (for expected counts < 5). Two-tailed *p* < 0.05 indicated significance.

Predictors with *p* < 0.10 in univariate logistic regression or known clinical relevance were considered for multivariable modeling. To prevent overfitting, the final model was restricted to three predictors based on the events-per-variable rule (∼10 events/predictor). Multicollinearity was ruled out using pairwise correlations (r < 0.7) and variance inflation factors (VIF < 2). HbA1c was modeled as continuous, and T2DM duration was dichotomized at ≥ 10 years.

Model discrimination was evaluated by the area under the receiver operating characteristic curve (AUC) with DeLong 95% confidence intervals; nested models were compared using DeLong's test and stratified bootstrap with 2,000 replicates. Calibration was assessed by the Hosmer-Lemeshow test. Internal validation with optimism-corrected AUC and calibration slope was performed with 1,000 bootstrap replicates. The study followed a consecutive-cohort design; all patients meeting the eligibility criteria during a fixed five-year enrollment window were included, without an *a priori* sample size calculation. With 26 outcome events and three predictors retained in the final multivariable model, the events-per-variable ratio was 8.67, approaching the widely recommended threshold of ten events per predictor (21). *Post-hoc* power analysis based on observed effect sizes indicated approximately 85% power at alpha = 0.05 to detect an odds ratio of 2.28 per 1% increase in HbA1c at *n* = 87 with an event rate of 29.9%, and moderate power (65 to 75%) to detect the observed effects of preoperative steatorrhea and T2DM duration.

## Results

**Figure 1:**
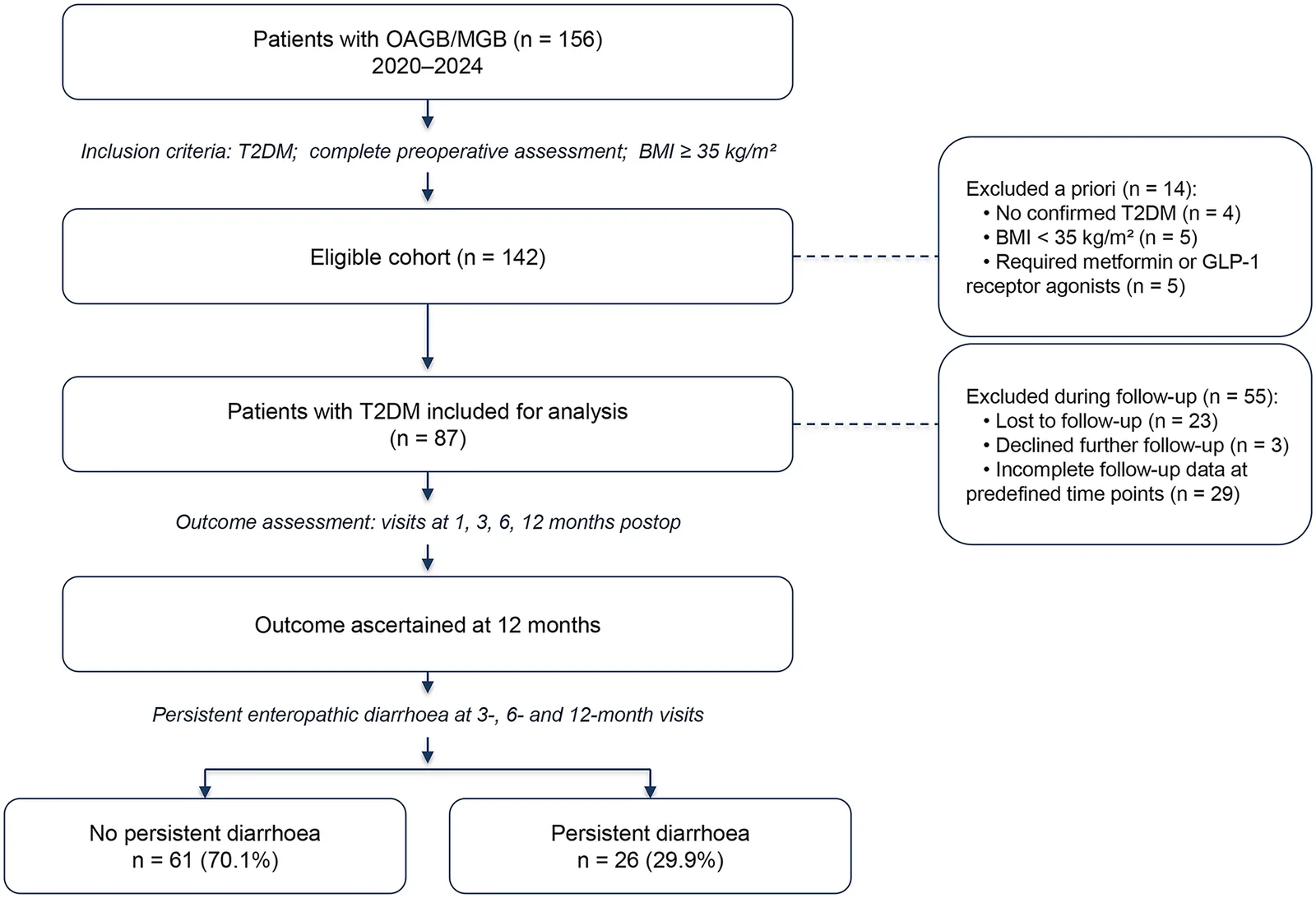
Flow of participants through the study (STROBE diagram).

Between January 2020 and December 2024, 156 patients underwent primary OAGB at our center; this constituted the initial screening pool for the present study. Of these, four did not have confirmed T2DM, five had a preoperative BMI below 35 kg/m^2, and five required metformin or a glucagon-like peptide-1 receptor agonist during the first postoperative year and were excluded *a priori* to prevent pharmacological confounding of the primary outcome. Of the remaining 142 eligible patients, 23 were lost to follow-up before the 12-month visit, three declined further follow-up, and 29 had incomplete follow-up data at one or more of the predefined assessment time points (1, 3, 6 or 12 months). The analysis cohort therefore comprised 87 patients (Figure 1). All 87 completed at least 12 months of follow-up with full outcome ascertainment; no patient was lost after the analysis cohort was defined. Preoperative coprogram, HbA1c and serum albumin were available for every patient; total protein and C-reactive protein were available for 87 and 67 patients, respectively. Postoperative weight loss at 12 months was substantial: mean total weight loss was 33.3 +/- 6.2% and mean excess weight loss was approximately 75 to 80%, consistent with published OAGB outcomes. Persistent enteropathic diarrhea was confirmed in 26 of 87 patients, an incidence of 29.9% (95% CI 20.5–40.6%). A further 22 patients had transient symptoms that resolved before the 12-month visit; they were not classified as having the primary outcome and were grouped with the 39 asymptomatic patients to form the comparator group (*n* = 61, 70.1%).

Baseline characteristics by outcome group are shown in Table 1. The two groups were similar in age (48.5 ± 8.8 vs. 51.1 ± 10.3 years, *p* = 0.255), sex distribution (female 78.7% vs. 73.1%, *p* = 0.771) and BMI [35.0 [IQR 32.0–40.0] vs. 34.8 [32.0–39.0] kg/m^2^, *p* = 0.515]. Patients who developed persistent diarrhea had higher preoperative HbA1c (9.60 ± 1.39% vs. 7.70 ± 1.50%, *p* < 0.001) and lower serum albumin (38.70 ± 4.53 vs. 41.23 ± 4.18 g/L, *p* = 0.019). Preoperative steatorrhea on coprogram was more than three times more common in the diarrhea group (50.0% vs. 14.8%, *p* = 0.001); mucus and undigested muscle fibers were also more frequent (30.8% vs. 6.6%, *p* = 0.005, and 42.3% vs. 14.8%, *p* = 0.005, respectively). T2DM duration of at least 10 years was recorded in 38.5% of the diarrhea group vs. 14.8% of the comparator group (*p* = 0.014). Fasting glucose, total protein, CRP, preoperative insulin therapy, hypertension, osteochondrosis and sleep apnea did not differ between groups (all *p* > 0.10; Table 1).

**Table 1:** Baseline demographic, clinical and laboratory characteristics of patients with T2DM undergoing OAGB.

Variable	No diarrhea (*n* = 61)	Persistent diarrhea (*n* = 26)	*p*-value
Demographic			
Age, years	48.5 ± 8.8	51.1 ± 10.3	0.255
Female sex, *n* (%)	48 (78.7)	19 (73.1)	0.771
Body mass index, kg/m2	35.0 (32.0–40.0)	34.8 (32.0–39.0)	0.515
Diabetes-related			
T2DM duration, years	5.0 (3.0–5.0)	5.0 (3.0–10.0)	0.489
T2DM duration ≥ 10 years, *n* (%)	9 (14.8)	10 (38.5)	**0.014**
Preoperative insulin therapy, *n* (%)	7 (11.5)	5 (19.2)	0.332
HbA1c, %	7.70 ± 1.50	9.60 ± 1.39	**<0.001**
Fasting glucose, mmol/L	7.63 ± 1.58	7.35 ± 1.80	0.506
Laboratory			
Serum albumin, g/L	41.23 ± 4.18	38.70 ± 4.53	**0.019**
Albumin ≤ 38.5 g/L, *n* (%)	17 (27.9)	14 (53.8)	**0.021**
Total protein, g/L	68.56 ± 4.46	69.44 ± 5.14	0.454
C-reactive protein, mg/L (available *n* = 45/*n* = 22)	8.8 (4.9–14.1)	6.0 (3.1–11.8)	0.109
Coprogram findings, *n* (%)			
Steatorrhea	9 (14.8)	13 (50.0)	**0.001**
Undigested muscle fibers	9 (14.8)	11 (42.3)	**0.005**
Mucus	4 (6.6)	8 (30.8)	**0.005**
Leucocytes	4 (6.6)	5 (19.2)	0.119
Comorbidities, *n* (%)			
Hypertension	36 (59.0)	18 (69.2)	0.369
Osteochondrosis	17 (27.9)	5 (19.2)	0.396
Sleep apnea	32 (52.5)	14 (53.8)	0.906

Values are mean ± SD, median (IQR) or *n* (%) as appropriate. Continuous variables compared by Welch's *t*-test (normally distributed) or Mann–Whitney *U* test (non-normal); categorical variables compared by Pearson's *χ*^2^ test or Fisher's exact test (when any expected cell count was < 5). Significant *p*-values (< 0.05) are shown in bold.

Preoperative variables associated with persistent enteropathic diarrhea at univariate logistic regression (*p* < 0.10) or of established clinical relevance were carried forward into the multivariable model. Univariate crude odds ratios and adjusted odds ratios from the final multivariable model are presented side by side in Table 2.

**Table 2:** Preoperative variables in relation to persistent enteropathic diarrhea at 12 months: crude (univariate) and adjusted (multivariable) odds ratios.

Predictor	Crude OR (95% CI)	*p*-value	Adjusted OR (95% CI)	*p*-value
HbA1c, per 1% increase	2.41 (1.65–3.86)	**<0.001**	2.28 (1.53–3.73)	**< 0.001**
Steatorrhea on coprogram, vs. absent	5.78 (2.07–17.02)	**0.001**	4.36 (1.21–16.82)	**0.026**
T2DM duration ≥ 10 years	3.61 (1.25–10.67)	**0.018**	3.90 (1.02–16.16)	0.050
Mucus on coprogram, vs. absent	6.33 (1.71–23.52)	**0.006**	—	—
Undigested muscle fibers, vs. absent	4.24 (1.48–12.13)	**0.007**	—	—
Serum albumin, per 1 g/L increase	0.87 (0.77–0.98)	**0.017**	—	—

Dashes (—) indicate variables that were not retained in the final multivariable model on the basis of prespecified selection criteria. Significant *p*-values (< 0.05) are shown in bold.

Model performance: AUC = 0.863 (95% CI 0.784–0.942); Hosmer–Lemeshow *χ*^2^ = 5.22, df = 6, *p* = 0.516; variance inflation factors < 1.10 for all predictors. DeLong's test for the difference in AUC between the combined model and HbA1c alone: *Δ*AUC = 0.037 (*p* = 0.21).

CI, confidence interval; HbA1c, glycated hemoglobin; OR, odds ratio; T2DM, type 2 diabetes mellitus.

The multivariable model was restricted to three predictors selected *a priori* on the basis of univariate evidence (*p* < 0.10), biological coherence and the absence of relevant multicollinearity. Among the coprogram items, steatorrhea was retained as the most clinically actionable marker of exocrine pancreatic function. Serum albumin was excluded because of its strong inverse correlation with steatorrhea (point-biserial r = −0.73), above the conventional threshold of (r) = 0.7 at which collinearity becomes problematic. T2DM duration was modeled as a binary variable (≥ 10 years) consistent with the dichotomy commonly used to define long-standing diabetes in the clinical literature. The final model therefore comprised HbA1c (continuous), steatorrhea (binary) and T2DM duration ≥ 10 years (binary). The events-per-variable ratio was 8.7 (26 events, three predictors), at the lower end of the conventionally accepted range (21), and variance inflation factors for all predictors were below 1.10, indicating no relevant residual collinearity.

**Figure 2:**
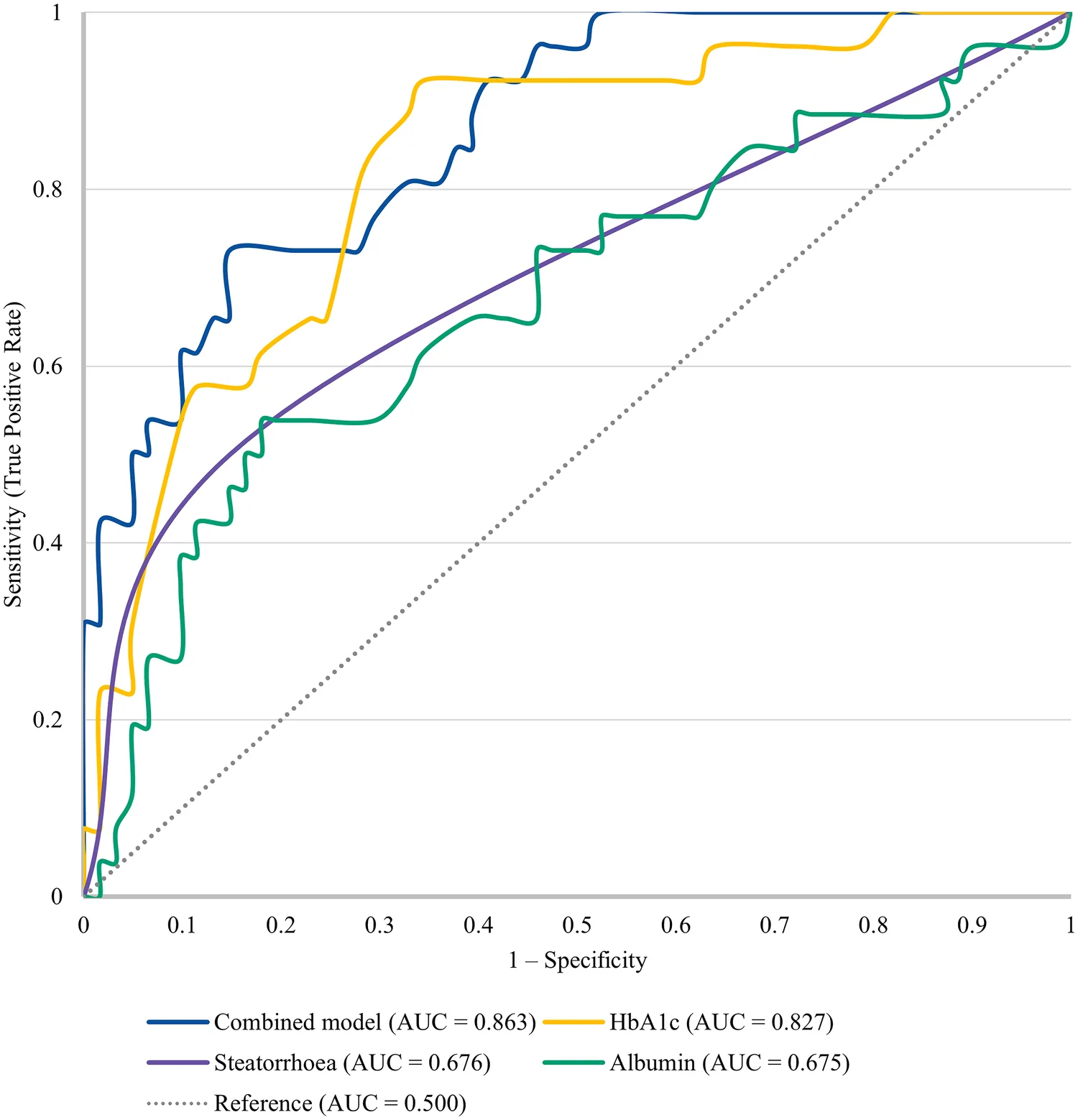
Receiver operating characteristic curves for the multivariable model (HbA1c + steatorrhea + T2DM duration ≥ 10 years) and for the individual predictors HbA1c, serum albumin and steatorrhea. The reference diagonal corresponds to a non-informative classifier (AUC = 0.500).

After mutual adjustment, all three predictors remained independently associated with the outcome (Table 2, adjusted odds ratio column). Each one-percentage-point increase in HbA1c was associated with an adjusted odds ratio (aOR) of 2.28 (95% CI 1.53 to 3.73, *p* < 0.001), preoperative steatorrhea on coprogram with an aOR of 4.36 (95% CI 1.21 to 16.82, *p* = 0.026), and T2DM duration of ten years or more with an aOR of 3.90 (95% CI 1.02 to 16.16, *p* = 0.050). The model showed good calibration (Hosmer-Lemeshow chi-squared = 5.22, df = 6, *p* = 0.516) and good discrimination, with an AUC of 0.863 (95% CI 0.784 to 0.942). Receiver operating characteristic curves for the combined model and for each individual predictor are shown in Figure 2. HbA1c alone provided an AUC of 0.827 (95% CI 0.733 to 0.920). The difference between the full model and HbA1c alone was not statistically significant (delta AUC 0.037, DeLong *p* = 0.21; bootstrap delta AUC 0.037, 95% CI −0.019 to 0.096). Internal validation with 1,000 bootstrap replicates produced an optimism-corrected AUC of 0.847 and a calibration slope of 0.893, indicating limited in-sample overfitting. Single-predictor receiver operating characteristic analyses identified Youden-optimal cut-off values for the two continuous predictors and for the combined model probability score (Table 3). For HbA1c, the optimal threshold was at or above 8.25%, with a sensitivity of 92.3%, a specificity of 65.6% and a negative predictive value of 95.2%.

**Table 3:** Receiver operating characteristic analysis: Youden-optimal cut-off values for individual predictors and for the multivariable model.

Predictor	Optimal cut-off	Sensitivity (%)	Specificity (%)	NPV (%)	PPV (%)	AUC (95% CI)
HbA1c	≥8.25%	92.3	65.6	95.2	53.3	0.827 (0.733–0.920)
Serum albumin	≤37.45 g/L	53.8	82.0	80.6	56.0	0.675 (0.554–0.796)
**Combined model**	***p*** **≥** **0**.**327**	**69**.**2**	**83**.**6**	**86**.**4**	**64**.**3**	**0.863** (**0.784–0.942)**

Cut-off values were derived using Youden's index. The combined model row is shown in bold to highlight its overall performance. Performance metrics were derived in the same cohort in which they were evaluated and are subject to optimism bias.

AUC, area under the receiver operating characteristic curve; CI, confidence interval; HbA1c, glycated hemoglobin; NPV, negative predictive value; PPV, positive predictive value.

**Figure 3:**
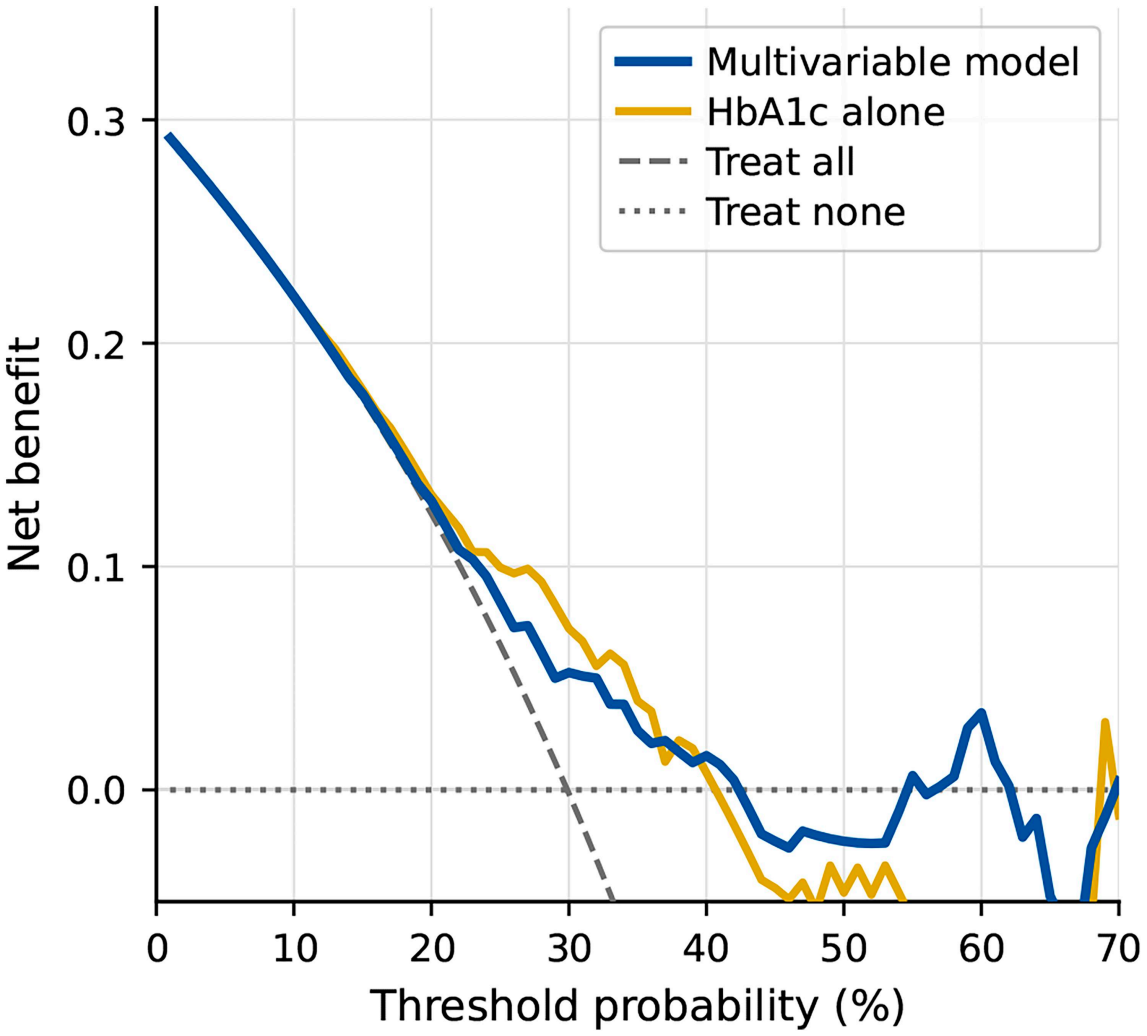
Decision curve analysis of the multivariable model and of HbA1c alone, compared with the reference strategies of treating all and treating no patients. Net benefit is plotted against threshold probability across the clinically relevant range.

Decision curve analysis showed net clinical benefit for both the multivariable model and HbA1c alone across threshold probabilities of approximately 10 to 60%, exceeding the default strategies of treating all patients or no patients. Consistent with the discrimination analysis, the net-benefit curves for the full model and for HbA1c alone were closely overlapping, supporting the use of preoperative HbA1c as a parsimonious single-variable decision rule within this range of clinically plausible risk thresholds (Figure 3).

In a sensitivity analysis stratified by protocolized biliopancreatic limb length, persistent enteropathic diarrhea occurred in 22 of 64 patients (34.4%) with a 180 cm limb and in 4 of 23 patients (17.4%) with a 200 cm limb (Fisher exact *p* = 0.185). No statistically significant association between protocolized limb length and the primary outcome was observed; the 200 cm subgroup is limited in size (*n* = 23), which constrains precision. Because biliopancreatic limb length was determined by BMI class per protocol, it was not entered as an independent covariate in the multivariable model (Supplementary Table S1).

## Discussion

In 87 consecutive patients with T2DM who underwent OAGB, persistent enteropathic diarrhea at 12 months developed in roughly three of every ten patients. Three preoperative variables, HbA1c, steatorrhea on coprogram and a T2DM duration of at least ten years, emerged as independent predictors of the outcome. Discrimination of the three-variable multivariable model (AUC 0.863) was not statistically superior to that of preoperative HbA1c alone (AUC 0.827; delta AUC 0.037, DeLong *p* = 0.21), and decision curve analysis showed closely overlapping net benefit for the two models across the clinically relevant threshold range (Figure 3). Preoperative HbA1c should therefore be regarded as the single most informative variable in this preoperative screening approach; steatorrhea on coprogram and diabetes duration of ten years or more provide complementary pathophysiological context and identify a high-risk phenotype for enhanced counseling and surveillance, but they add limited discriminative information beyond HbA1c. The association between preoperative HbA1c and persistent enteropathic diarrhea, a 2.3-fold increase in adjusted odds per 1% rise, is biologically plausible. Chronic hyperglycemia is associated with progression of diabetic autonomic neuropathy and with impaired intestinal barrier function; both mechanisms may plausibly contribute to persistent postoperative diarrhea by predisposing to altered enteric motility, small intestinal bacterial overgrowth and reduced mucosal integrity (14, 15). Our observation is consistent with, but does not demonstrate, a causal role for chronic hyperglycemia; it should be interpreted as an association whose mechanistic basis warrants confirmation in prospective studies incorporating objective mechanistic testing.

Preoperative steatorrhea on coprogram was the strongest categorical predictor in our cohort, with an adjusted odds ratio of 4.4. This finding is compatible with earlier reports of under-recognized subclinical exocrine pancreatic insufficiency in patients with T2DM. Earlier work in T2DM cohorts has documented low fecal elastase-1 levels in a substantial proportion of patients even in the absence of overt gastrointestinal symptoms, and reduced pancreatic exocrine reserve may become clinically evident after OAGB, when the shortened common channel further limits enzyme-substrate contact (10, 13). Because fecal elastase and other objective tests of pancreatic exocrine function were not performed in the present cohort, this pathophysiological link remains hypothetical and should be tested prospectively. The association between long-standing T2DM (ten years or more) and persistent enteropathic diarrhea is consistent with a cumulative-injury model of diabetic enteropathy, in which longer disease duration serves as a marker of accumulated neural and microvascular damage. Longer disease duration is also associated with a higher likelihood of subclinical exocrine pancreatic insufficiency, providing a plausible pathophysiological convergence between two of our three independent predictors. As with the HbA1c and steatorrhea associations, however, mechanistic interpretation remains hypothetical in the absence of objective testing and should be confirmed in dedicated prospective studies. Serum albumin was significantly associated with diarrhea in univariate analysis but was not retained in the final multivariable model because of its strong inverse correlation with steatorrhea. This pattern is consistent with low preoperative albumin acting as a surrogate marker of subclinical maldigestion and chronic inflammation rather than as an independent causal driver. The clinical message is unchanged: a low preoperative albumin remains an easily measured warning sign, and the absolute risk increase associated with values at or below 37.45 g/L is similar in magnitude to that reported in the much larger registry analysis of McLean et al. (16).

A number of our findings are notable for what they did not show. CRP, despite a strong biological rationale and a generally elevated median in our cohort, was not associated with diarrhea in either univariate or sensitivity analyses. The most likely reason is the relatively narrow range of CRP values in a T2DM-only cohort, which limited the contrast between groups. Preoperative insulin therapy and the common obesity-related comorbidities — hypertension, sleep apnea and osteochondrosis — also showed no association. These negative findings argue against the use of comorbidity counts or insulin status as substitutes for the direct biochemical and stool-based markers we identified.

The observed rate of persistent enteropathic diarrhea in our cohort (29.9%, 95% CI 20.5 to 40.6%) is at the upper end of reported ranges for OAGB and higher than most rates reported after Roux-en-Y gastric bypass or sleeve gastrectomy. Published OAGB series report persistent diarrhea in approximately 15 to 25% of general OAGB cohorts, rising to over 30% when the biliopancreatic limb is 200 cm or longer. Our slightly higher rate is most plausibly explained by the enriched T2DM composition of our cohort (100%, vs. typically 25 to 40% in general bariatric series), consistent with the hypothesized contribution of subclinical diabetic enteropathy to postoperative gastrointestinal dysfunction.

These findings translate into a simple preoperative screening approach with immediate clinical utility. A patient with preoperative HbA1c below 8.25% has an estimated absolute risk of persistent enteropathic diarrhea of approximately 5% (negative predictive value 95.2%) and can be reassured. A patient at or above this threshold, particularly one with concurrent steatorrhea on coprogram or diabetes of more than a decade, warrants enhanced preoperative counseling regarding the risk of persistent postoperative diarrhea, consideration of the shorter biliopancreatic limb where BMI allows, and structured postoperative surveillance for treatable causes such as small intestinal bacterial overgrowth, bile acid malabsorption, and pancreatic exocrine insufficiency. Because HbA1c, coprogram, and diabetes duration are already collected in routine bariatric workup, no additional investigations or costs are incurred.

Several limitations should be acknowledged. First, this was a single-center consecutive cohort without an *a priori* sample size calculation. With 26 outcome events and three predictors, the events-per-variable ratio was 8.67, approaching but not reaching the recommended threshold of ten (Peduzzi et al., 1996); confidence intervals for the less-prevalent predictors (steatorrhea on coprogram and T2DM duration of ten years or more) were wide, and bootstrap resampling addresses only internal, not external, validity. The optimism-corrected AUC of 0.847 and calibration slope of 0.893 indicate limited in-sample overfitting, but the findings should be regarded as exploratory and hypothesis-generating until prospectively validated in an independent, adequately powered cohort.

Second, the primary outcome was defined by clinical documentation of stool character at three structured follow-up visits and was not measured with the Bristol Stool Form Scale or a validated gastrointestinal quality-of-life instrument. The requirement of concordant documentation at three time points reduces single-visit misclassification, but incorporation of the Bristol scale and a validated instrument such as the GIQLI or IBS-QoL will strengthen outcome ascertainment in prospective replication studies.

Third, no objective mechanistic testing (fecal elastase for pancreatic exocrine insufficiency, glucose or lactulose breath testing for small intestinal bacterial overgrowth, SeHCAT or 7-alpha-hydroxy-4-cholesten-3-one for bile acid malabsorption) was performed. These investigations are not part of routine preoperative or postoperative bariatric workup at our institution during the study period, consistent with practice at most bariatric centers where such testing is reserved for symptomatic postoperative patients rather than performed prospectively for research purposes. Similarly, total intestinal length was not measured intraoperatively, and individual dietary adherence was assessed only qualitatively. The pathophysiological mechanisms invoked in the Discussion are therefore hypothetical, and future prospective studies should combine preoperative risk stratification with objective mechanistic workup and quantitative adherence measures.

In patients with T2DM undergoing one-anastomosis gastric bypass, preoperative HbA1c is a robust, clinically accessible single-variable predictor of persistent enteropathic diarrhea at 12 months. Steatorrhea on coprogram and long-standing diabetes add complementary pathophysiological context but do not significantly improve discrimination beyond HbA1c alone. These routine preoperative metrics can inform patient counseling and risk-adapted postoperative surveillance and warrant prospective external validation in independent cohorts.

## Data Availability

The raw data supporting the conclusions of this article will be made available by the authors, without undue reservation.
