# Supplementary material for: Preoperative predictors of persistent enteropathic diarrhea after one-anastomosis gastric bypass in patients with type 2 diabetes mellitus
Source: Front Surg. 2026 Aug 12;13:1901971. doi: 10.3389/fsurg.2026.1901971 (PMC13506704; doi:10.3389/fsurg.2026.1901971)
Supplement: Supplementary file 1 [file Datasheet1.pdf]

Supplementary Material

Supplementary Table 1. Event rate of persistent enteropathic diarrhea by protocolized biliopancreatic limb length.

Biliopancreatic limb length	No diarrhea (n)	Persistent diarrhea (n)	Total (n)	Event rate (%)
**180 cm (BMI 35–39.9 kg/m²)**	42	22	64	34.4
**200 cm (BMI ≥ 40 kg/m²)**	19	4	23	17.4
**Total**	**61**	**26**	**87**	**29.9**

Biliopancreatic limb length was standardized by BMI class per institutional protocol (180 cm for class II obesity, 200 cm for class III–IV obesity). Fisher exact test comparing event rates between the two limb-length groups: p = 0.185. Because biliopancreatic limb length was deterministically defined by BMI class, it was not entered as an independent covariate in the multivariable model.
